# Quorum Sensing Regulation of Competence and Bacteriocins in *Streptococcus pneumoniae* and *mutans*

**DOI:** 10.3390/genes8010015

**Published:** 2017-01-05

**Authors:** Erin Shanker, Michael J. Federle

**Affiliations:** Department of Medicinal Chemistry and Pharmacognosy, University of Illinois at Chicago, Chicago, IL 60607, USA; ebeisn2@uic.edu

**Keywords:** quorum sensing, competence, transformation, bacteriocin, fratricide, gene regulation

## Abstract

The human pathogens *Streptococcus pneumoniae* and *Streptococcus mutans* have both evolved complex quorum sensing (QS) systems that regulate the production of bacteriocins and the entry into the competent state, a requirement for natural transformation. Natural transformation provides bacteria with a mechanism to repair damaged genes or as a source of new advantageous traits. In *S. pneumoniae*, the competence pathway is controlled by the two-component signal transduction pathway ComCDE, which directly regulates SigX, the alternative sigma factor required for the initiation into competence. Over the past two decades, effectors of cellular killing (i.e., fratricides) have been recognized as important targets of the pneumococcal competence QS pathway. Recently, direct interactions between the ComCDE and the paralogous BlpRH pathway, regulating bacteriocin production, were identified, further strengthening the interconnections between these two QS systems. Interestingly, a similar theme is being revealed in *S. mutans*, the primary etiological agent of dental caries. This review compares the relationship between the bacteriocin and the competence QS pathways in both *S. pneumoniae* and *S. mutans*, and hopes to provide clues to regulatory pathways across the genus *Streptococcus* as a potential tool to efficiently investigate putative competence pathways in nontransformable streptococci.

## 1. Introduction

Nearly 100 years have passed since the first hints of bacterial communication were observed in Griffith’s seminal descriptions of what came to be understood as horizontal gene transfer in *Streptococcus pneumoniae* [1]. However, the study of quorum sensing (QS) and the molecular mechanisms underlying coordinated processes leading to the development of competence and the production of bacteriocins (bacterially-derived antimicrobial proteins) has taken the decades since to uncover the signals and the regulatory pathways required for these processes. Unraveling the intricacies of a particular QS system is crucial for the development of methods to modify downstream bacterial behavior. Although great progress has been made in describing bacterial quorum sensing pathways, it is apparent that the complexities of each pathway are not thoroughly understood. This review compares recent findings describing the regulation of competence and the direct killing mechanisms of *Streptococcus pneumoniae* and *Streptococcus mutans*.

## 2. A Brief Introduction to Quorum Sensing

Quorum sensing is a method of communication employed by bacteria to coordinate a response amongst a population. This ability is shared between both Gram-negative and Gram-positive bacteria (for a recent review of each, see Papenfort and Bassler [2], and Monnet et al. [3], respectively). Briefly, bacterially derived signals, referred to as pheromones, are excreted by bacteria and sensed, in a concentration-dependent manner, by neighboring bacterial cells to elicit a response. These signals can be small molecules, such as acyl-homoserine lactone derivatives, commonly employed by Gram-negative bacteria, or small peptides, generally preferred by Gram-positive bacteria (reviewed by Johnston et al. [4]). Since this process is dependent upon the concentration of signals, any condition that influences pheromone concentration (for example, signal degradation, diffusion, or sequestration) will impact the ability of cells to transmit information. The coordinated responses generated by pheromones regulate a wide variety of physiological processes ranging from the expression of virulence factors, a function the PapR/PlcR and NprX/NprR QS systems control in *Bacillus*; to the formation of biofilms, in part, modulated by the SHP/Rgg circuit in *Streptococcus pyogenes*; and the entrance into the competent state, governed by the XIP/ComR pathway in *S. mutans* (recently reviewed by Perez-Pascual et al. [5]).

Competence, a prerequisite of natural transformation, is the capacity of cells to take up extracellular DNA (eDNA) and incorporate it into their genome (see Johnston et al. [4]). In this way, cells capable of natural transformation can use eDNA to repair damaged DNA or, alternatively, to acquire new traits, such as antibiotic resistance or the expression of a novel toxin. The nature of the eDNA limits the usefulness to the acquiring cell because homologous recombination is necessary for a successful integration event. Therefore, the more closely related the eDNA is to the genome of the competent cell, the more likely a productive recombination event will occur (reviewed by Mell and Redfield [6]). This review focuses on the strategies that *S. pneumoniae* and *S. mutans* use to ensure access to related eDNA, a tactic that appears to have co-evolved with the ability to lyse neighboring species and/or strains residing within the same microbial niche.

## 3. The Competence Pathway in *Streptococcus pneumoniae*

**Figure 1:**
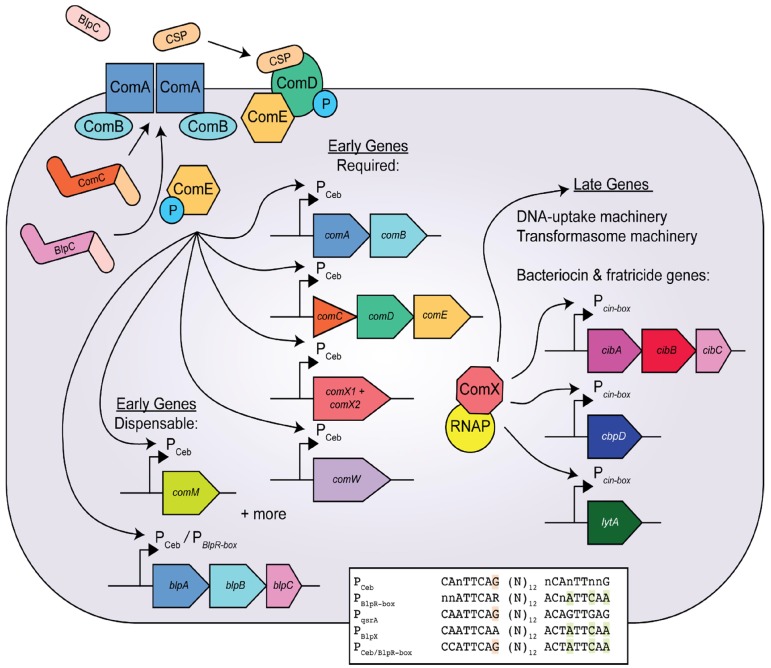
The ComABCDE quorum sensing (QS) pathway of *Streptococcus pneumoniae*. The two phases of competence development are controlled by ComE, for induction of early genes, and by ComX, for induction of late genes. Early genes required for competence include *comE*, *comAB*, *comCDE*, *comX1*/*comX2*, and *comW*. Dispensable early genes include *comM* as well as *blpABC*, and *blpXYZ* [20]. Phosphorylated ComE binds at P_Ceb_, a promoter site containing a ComE binding site (Cbe) [14,16,21]. Wholey et al. [33] recently demonstrated dual regulation of the *blpABC* operon either by ComE or BlpR in *S. pneumoniae* (denoted by P_Ceb_/P*_BlpR-box_*) [10]. The alignment of the upstream sequence of ComE- and BlpR-regulated genes are shown in the figure inset. The consensus sequences for P_Ceb_- and P*_BlpR-box_*-direct-repeat binding motifs in the *S. pneumoniae* TIGR4 genome are compared with the upstream sequences of *qsrA* (locus ID: SP_1717), *blpX* (locus ID: SP_0544) and the direct-repeat hybrid motif (P_Ceb/BlpR-box_) located upstream of *blpA* (locus ID: SP_0530). Highlighted in orange is the guanine base pair that is conserved in ComE-regulated promoters, permitting ComE to regulate expression of the *blpABC* [33]. Highlighted in green are base pairs conserved in the proximal motif of BlpR-regulated promoters. This sequence alignment has been adapted from Wholey et al. [33]. The late genes are directly regulated by the ComX/RNAP (RNA polymerase) complex and consist of genes required for DNA uptake, homologous recombination as well as the lytic genes, *cibABC*, *cbpD*, and *lytA*, encoding effectors of fratricide [20]. The ComAB complex exports and processes ComC into the mature peptide pheromone CSP [10]. Recently, ComAB was demonstrated to export and process BlpC. This connection between the bacteriocin and competence QS system pathways clarifies how BlpC activity is detected in strains lacking a functional BlpC exporter (BlpA) [33,34].

The study of natural transformation in bacteria began after Frederick Griffith observed the transfer of morphological traits between different strains of *S. pneumoniae*. This transfer of phenotypes was later shown to be the result of DNA exchange regulated by a secreted peptide pheromone, the competence-stimulating peptide (CSP) [7]. The precursor peptide of CSP, ComC, contains an N-terminal leader peptide that shares homology with leader peptides found in the class II family of unmodified bactericidal peptides [8,9]. This leader sequence also contains a double-glycine motif that directs the pre-peptide for export and processing through an ABC transporter/protease, ComAB [7,9,10]. During export, ComC is cleaved by ComAB immediately after the double-glycine motif to yield the active CSP [10]. No other modifications to CSP are known to occur in *S. pneumoniae*, thereby distinguishing CSP from the analog mutacin-inducing peptide (MIP) (see Table 1) in *S. mutans*, which is further processed extracellularly by the protease SepM [11]. Extracellular CSP then binds and activates the cognate histidine kinase receptor ComD of the ComCDE two-component signal-transduction pathway (TCST). Activation of ComD results in autophosphorylation and the subsequent transfer of a phosphoryl group (~P) to the cytosolic response regulator ComE [12,13,14]. Phosphorylated ComE (ComE~P) binds to a conserved promoter sequence, referred to as a ComE-binding site, or Ceb, found upstream of the alternative sigma factor gene *sigX*/*comX*, for which there are two identical genomic copies within the *S. pneumoniae* genome [14,15,16]. *comX* activity is essential for the expression of many genes required for competence (for recent reviews see [4,17,18]) [14,15,19,20]. This conserved Ceb element is also found near the *comAB* operon as well as the *comCDE* operon. ComE~P binds at the Ceb in the promoter region (P_Ceb_) of these operons, among others, to activate gene expression, resulting in the amplification of the core components of the CSP signal transduction pathway [21,20] (Figure 1).

The induction of competence in *S. pneumoniae* is divided into two temporally distinct phases, the early and the late, regulated by ComE~P or ComX, respectively [19,20]. Besides activating transcription of *comX*, *comAB*, and *comCDE*, ComE~P also activates transcription of at least 17 other genes, several of which are required for the development of the competent state [20]. The early gene (defined by the maximal expression between 7.5 and 10 min post-CSP treatment), *comW*, is important in natural transformation as it is required for stabilizing SigX protein levels and enabling SigX activity by way of an unknown mechanism [20,35,36,37]. Early genes observed to contribute to transformation, yet are dispensable, include *comM*, a membrane protein that imparts cellular immunity towards lytic enzymes as well as *blpAB* which encodes a bacteriocin transporter that shares homology with the CSP exporter, *comAB* [38,39,40] (Figure 1).

The expression levels of ComX-induced late genes peak approximately 12.5 to 15 min post-CSP induction [20]. Although the late genes comprise over 80 genes, only 14 have been identified as essential for transformation [20]. These required genes include those encoding the DNA-uptake machinery and the recombination machinery (also, referred to as the transformasome (see Claverys et al. [41] for a review) (Figure 1).

## 4. Bacteriocin Regulation and Function in *S. pneumoniae*

Entry into the state of competence is an energetically costly endeavor, resulting in the coordinated expression of over 100 CSP-responsive genes [20]. For *S. pneumoniae* to benefit from a state of competence, a source of “naked”, or extracellular DNA must also be available. This DNA could be derived from closely related strains, more distantly related species, or even from virtually identical sibling cells. Therefore, it is not surprising that *S. pneumoniae* has evolved mechanisms to ensure access to DNA. One such mechanism, termed allolysis or fratricide, results from the targeted lysis of non-competent *S. pneumoniae* cells by their competent siblings, hence the term fratricide [42,43].

At least six genes implicated in fratricide are up regulated in *S. pneumoniae* in response to CSP stimulation. These include the previously mentioned early gene *comM*, as well as *cibABC*, *cbpD*, and *lytA*, all of which are induced by ComX alongside other late competence genes [20]. CibAB consists of a two-peptide bacteriocin responsible for lysis of cells lacking the corresponding immunity factor, CibC [44]. Bacteriocins are a class of small, ribosomally-synthesized peptides that exhibit antimicrobial activity towards bacteria that lack the corresponding immunity factor [45]. Therefore, a competent *S. pneumoniae* cell would express CibABC and would then be capable of targeting a nearby non-competent *S. pneumoniae* cell that is unable to express functional CibC. Generally, type II bacteriocins, including CibAB, are thought to exert cidal effects on target cells through mechanisms ultimately leading to membrane leakage. For example, type IIa bacteriocins, representing a subclass of pediocin-like linear un-modified peptides, bind to the mannose-phosphotransferase system (Man-PTS) on a target cell, initiating the formation of a pore [46] (recently reviewed by Alvarez-Sieiro [47]). CbpD is a murein hydrolase containing a cytosine, histidine-dependent amidohydrolase peptidase (CHAP) domain that functions extracellularly to degrade murein stem peptides found in the pneumococcal cell wall, resulting in structural changes that promote lysis [44]. Competence-induced expression of the early gene *comM*, encoding a membrane-bound protein, confers immunity to CbpD-mediated lysis [39]. For fratricide to occur in liquid culture, full CbpD activity requires LytA, an effector of autolysis in *S. pneumoniae* [42,43,48]. Furthermore, a separate lytic amidase, not known to be regulated by competence, LytC, is also required for complete competence-induced fratricide [44].

Interestingly, both of these mechanisms of predation appear to be restricted to isogenic or closely related strains [49,50]. This observation fits with the suggestion that competent cells target related cells to acquire homologous DNA sequences to either maintain genome integrity or to acquire new gene alleles from the pneumococcal meta-gene pool to provide phenotypic heterogeneity and diversity that could enhance fitness within the community. The likelihood for transformation correlates directly with increasing homology between the donor’s DNA and the recipient’s genome since success relies on homologous recombination [6]. However, *cibABC*/*cbpD*/*lytA*-dependent transformation may not entirely account for the apparent extensive horizontal gene transfer present in pneumococcus.

## 5. A Second Bacteriocin QS System Cryptically Linked to Competence in *S. pneumoniae*

Recently, new evidence has demonstrated that a separate bacteriocin QS pathway may contribute to the acquisition of DNA by competent pneumococcal cells [33,34]. As previously mentioned, when Peterson et al. [20] originally described the temporally distinct gene sets activated post-CSP induction, a bacteriocin-like peptide (blp) ABC transporter, *blpAB*, was found to be among the early genes. Curiously, there is no clear Ceb-containing promoter site located upstream of this locus. Additionally, oriented in the opposite direction but directly adjacent to this locus is a pair of putative bacteriocin immunity genes, *blpY* and *blpZ*, that were also observed to be early genes, also without an obvious upstream Ceb element [20]. 

Although these loci had not yet been studied in relation to competence in *S. pneumoniae* at the time, they had been observed to be regulated by the BlpRH TCST QS pathway, which is implicated in virulence in a mouse infection model [51]. Upon detailed inspection, de Saizieu et al. [38] noted the high degree of similarity between the streptococcal *blpAB* and *comAB* sequences (65% and 31% identity is shared between the A and B subunits, respectively) [52]. Significantly, directly downstream of *blpAB*, there is a small open reading frame (ORF), now termed *blpC*, encoding a double-glycine motif and possessing leader peptide homology to the family of class II bacteriocin peptides, which also includes CSP [8,38]. Intriguingly, only ~20%–30% of all sequenced *S. pneumoniae* isolates encode a (predicted) functional *blpAB* locus [34,52].

Further characterization of the Blp pathway revealed additional similarities to the ComABCDE pathway that regulates competence. For example, mature BlpC peptide pheromone was found to activate the histidine kinase receptor, BlpH, which in turn resulted in phosphorylation of the response regulator BlpR, paralleling the signaling mechanism of CSP [38]. Among the genes up-regulated upon BlpC induction are *blpABC*, *blpXYZ*, and *blpSRH*, as well as several putative bacteriocins and related immunity proteins that constitute the Bacteriocin Immunity Region (BIR) [38,53,54]. Analysis of the major BlpC-induced transcripts resulted in the identification of a highly conserved consensus promoter element predicted to bind activated BlpR. Upon inspection of the *S. pneumoniae* genome, no additional putative BlpR promoter sites were identified, even in regions upstream of the genes known to directly regulate competence [38]. This observation, along with the finding that *blpA* is not essential for transformation under laboratory conditions, hindered the connection between this bacteriocin pathway and the CSP-regulated competence [20,38]. 

In 2004, Knutsen et al. [40] described the promoter region of an early nonessential competence locus encoding a putative ATP transporter, *qsrAB*, that is induced upon treatment with CSP or with BlpC. Induction relied on the transcriptional regulators ComE and BlpR, suggesting that either factor could induce *qsrAB* [54]. By comparing the upstream regulatory region of *qsrAB* with the conserved ComE-regulated promoter, Knutsen and colleagues characterized the *qsrAB* promoter as having a “hybrid direct repeat motif” where the distal motif shares more homology to the conserved *blpR*-regulated promoters and the proximal motif is more similar to the Ceb motif [40] (see Figure 1, inset). By mutating the *comE*-like motif to be more similar to the *blpR* one, they observed an increased induction of *qsrAB* by BlpC treatment [40]. This observation that a hybrid direct-repeat promoter could accommodate separate transcriptional regulators, each with its own distinctive target motif, facilitated the discovery that bacteriocin and competence pathways are more closely linked than previously thought.

## 6. A Direct Link between *blp* and *com* Is Established in *S. pneumoniae*

Recently, two groups independently uncovered direct links between the ComABCDE and the BlpABC/BlpRH pathways. As previously mentioned, ~70%–80% of all sequenced pneumococcal isolates encode a non-functional BlpAB transporter [34,52]. Kjos et al. [34] asked whether this degeneracy was correlated to gene loss within the BlpR-regulated BIR as a result of genetic drift. Interestingly, compared to genomes containing an intact *blpAB* locus, there were no significant differences in the number of predicted bacteriocins encoded within the *blpAB*-deficient strains [24]. Experiments performed in a strain lacking a functional BlpAB transporter demonstrated natural induction of *blp* loci during culture growth in a competence-permissive medium (C + Y, ≥7.4 pH) or upon exposure to sub-lethal concentrations of the antibiotics HPUra (6-(*p*-hydroxyphenylazo)-uracil), ciprofloxacin, and streptomycin. These induction profiles closely mimicked expression patterns previously associated with the ComE-regulated genes under identical conditions [34,55,56,57].

Similarly, Wholey et al. [33] demonstrated induction of *blp* operons in response to exogenous CSP, even when the *comAB* locus was knocked out. They also showed that this effect required intact *comE*, *blpC*, and *blpH*. Furthermore, this group observed decreased levels of secreted BlpC in the supernatants of strains lacking a ComAB transporter. As both BlpC and ComC pre-peptides encode an N-terminal leader peptide containing a double-glycine motif, this result suggested that perhaps the ComAB transporter was able to export and process BlpC as well as ComC [33]. Indeed, both Wholey et al. and Kjos et al. [33,34] demonstrated that ComAB was responsible for the processing and exporting of pre-BlpC in a *blpA* deficient mutant (Figure 1). However, the levels of matured BlpC detected in whole-cell lysates suggested that only ~30% of the pre-BlpC peptide could be exported by ComAB when compared to a *comAB*/*blpA* sufficient strain [33].

The previous finding by Knutsen et al., [40] that the hybrid direct-repeat motifs within the promoter region of the early genes *qsrAB* could accommodate both ComE and BlpR, led Wholey et al. to probe this same possibility as an explanation for the CSP-induced expression of the *blpABC* locus. When the distal motif of the *blpR*-box direct repeat upstream of *blpABC* was aligned with the corresponding region of the *comE*, *qsrA* distal motifs, Wholey and colleagues identified a single base pair that was conserved in the *blpA* (CSP-inducible) motif, but not in the other BlpR-regulated motifs (Figure 1, inset). After mutating this base pair to that of the corresponding base pair found in all other BlpR-regulated motifs, Wholey et al. observed a loss of CSP-induced BIR expression [33]. Furthermore, BlpC-induced expression of the BIR remained intact, suggesting that this direct repeat could no longer accommodate ComE binding, yet still retained the ability to bind BlpR [33]. The co-evolution of competence development and bacteriocin production not only strongly implicates their parallel functions, but also suggests that the interconnection between these mechanisms provides an advantage in predation on other cells. Interestingly, a similar relationship is emerging between these two QS pathways in the oral pathogen *S. mutans*, suggesting a potentially conserved theme among competent *Streptococcus*.

## 7. The Multiple Layers of Competence Regulation in *Streptococcus mutans*

An apparent homolog of the ComCDE system of *S. pneumoniae* identified in *S. mutans* modulates competence [26]. Upon addition of the putative CSP to *S. mutans* growing in peptide-rich media (i.e., Todd–Hewitt Broth (THB)), SigX activation and competence was observed, resulting in the designation of these genes as *comCDE* in *S. mutans* [26]. However, as mentioned earlier, the *S. pneumoniae* genome encodes an additional *comCDE*-like locus, *blpABC/RH*, as a regulatory system that controls production of bacteriocin-like peptides [38,58]. In fact, the *S. mutans* genes originally designated as *comCDE* shares a higher degree of sequence homology to the *S. pneumoniae blp* locus than it does with the *S. pneumoniae comCDE* locus [29] (Table 1). It was proposed by Hale et al. [27] that *S. mutans comA* and *comB* be renamed *nlmT* (non-lantibiotic mutacin transporter) and *nlmE* (*nlmT* accessory protein), respectively, to better reflect these genes products as bacteriocin-related in function.

Although the ComCDE QS pathway in *S. pneumoniae* and the homologous *blpHR* pathway in *S. mutans* seemingly perform the same function of activating competence, differences in locus arrangement and gene conservation [29], kinetics of competence development [7,19,59], and requirements (or lack thereof) of *S. mutans* “ComCDE” components for transformation [26] together suggest that *S. mutans* harbors an alternative pathway more directly responsible for SigX activation than the apparent homolog of *comCDE* [29,31]. The identification of an alternative regulatory pathway uncovered in the Salivarius group of *Streptococcus* provided a likely candidate [60]. Using a transcriptomics approach, Fontaine et al. [60] identified a peptide pheromone, ComS, encoded directly downstream of an Rgg-like transcriptional regulator, ComR, that proved to be required for SigX activity. An orthologous *comRS* locus in *S. mutans* was subsequently identified and found to function similarly in activating competence by controlling *sigX* transcription [31].

**Figure 2:**
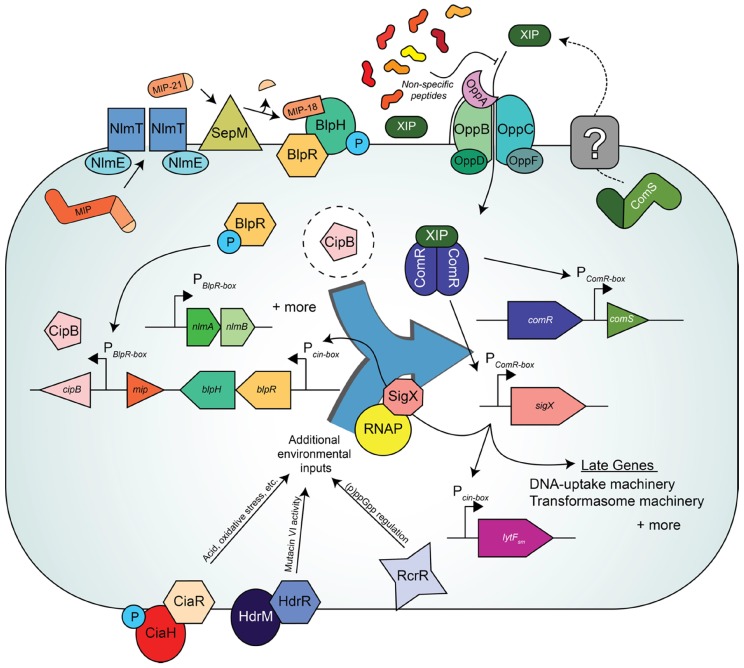
The ComRS quorum sensing (QS) pathway controls competence, and the BlpRH QS system regulates bacteriocin production in *S. mutans* strain UA159. The ComS pre-peptide is exported and processed via an unknown mechanism (denoted as a dotted line and gray box) into XIP. In a chemically defined, peptide-poor medium (CDM) XIP is re-imported via oligopeptide permease (Opp) and binds ComR. The ComR/XIP complex binds upstream of *comS* and *sigX* at P*_ComR_*-box sites [31]. The alternative sigma factor, SigX, facilitates RNA polymerase (RNAP) binding at P*_cin-box_* sites [64]. Genes regulated by SigX include the DNA-uptake and transformasome machinery as well as the murein hydrolase, LytF*sm*, among others [59,68]. Non-specific peptides present in high concentrations in peptide-rich media are thought to prevent intracellular transport of XIP via Opp [69]. Recently, a link between the bacteriocin and competence pathways was determined to be mediated by SigX at a P*_cin-box_* site upstream of *blpRH* (formerly referred to as *comDE*, see Table 1). The BlpRH QS pathway pheromone, MIP, is exported and processed by the ABC transporter complex NlmTE into the 21 amino-acid long precursor peptide, MIP-21 [27]. Cleavage of MIP-21 by the protease SepM results in fully active MIP-18 [11]. MIP-18 is thought to bind to BlpH, resulting in activation and phosphorylation of BlpR similarly to CSP binding and activation of ComDE in *S. pneumoniae* [13]. BlpR~P binds at P*_BlpR-box_* sites upstream of bacteriocin loci such as *cipB* and *nlmAB*, among others [70]. Although the mechanism is unknown (represented by the dotted circle surrounding CipB), the activity of CipB is required for the indirect induction of SigX observed when cells are grown in peptide-rich media and treated with MIP [22,25,71]. Multiple other regulatory pathways are observed to feed into the ComRS/BlpRH QS networks, such as the CiaHR, HdrRM, and the RcrRPQ systems [72,73,74,75,76,77]. These additional inputs along with possible contribution through the BlpRH-regulated QS pathway (i.e., via *cipB* expression [71]) may help *S. mutans* to integrate environmental cues into the competence signaling cascade, allowing for fine tune control of natural transformation (depicted by the blue block arrow).

For *S. mutans*, it was proposed that the pre-peptide ComS is exported to the extracellular space and processed into the active seven amino acid residue peptide pheromone, SigX-inducing peptide (XIP), by an unknown mechanism [31,61]. In support of this model that XIP exists outside the cytoplasm, competence-inducing activity was retrieved from cell-free culture supernatants [62], the seven-residue peptide was identified by mass-spectrometry [61], and the oligopeptide permease, Opp/Ami, was required for activity, at least in CDM [31]. It is reasoned that XIP accumulates and is subsequently imported until concentrations of the peptide reach the level necessary to bind cytosolic ComR [31]. Upon binding XIP, ComR dimerizes and binds to a conserved inverted repeat (IR) sequence directly upstream of both *comS* and *sigX*, resulting in auto-induction of the ComRS pathway and the activation of the SigX regulon, respectively [31,63]. Similar to the induction of late genes in *S. pneumoniae*, the genes directly regulated by SigX are characterized by the separate conserved motif required for SigX binding, the cin-box ([64]) (Figure 2).

It is worth noting that although the Salivarius ComRS pathway led to the discovery of the Mutans ComRS equivalent, they differ in multiple aspects, resulting in the categorization of each separately as the Type-I and Type-II ComRS systems, respectively [31,60]. Briefly, they differ in locus arrangement in the genome and in the conserved P*_comS_*/P*_sigX_* promoter sequences (P*_comR_*_-box_), but with potentially more significance they differ in the composition of the active XIP sequences and in the requirement for processing by the protease Eep (Eep is not required for Type-II ComS maturation) [31,60,61,63,65]. These latter characteristics indicate that pheromone biosynthesis and maturation follow different paths and therefore may have different physical properties that could impact signal dispersion, longevity, or specificity within complex biological systems, including among diverse microbial populations. Regardless of such differences however, ComRs from each group directly bind their respective XIPs to activate competence. Insights into the precise molecular interactions between receptors and ligands were demonstrated recently by structural and genetic analysis [66,67]. 

## 8. How Does Peptide-Rich Versus Peptide-Poor Medium Affect Competence and Bacteriocin QS Pathways?: “The Medium Is the Message”*—Marshall McLuhan* [78]

Just as the relationship between the regulation of competence and bacteriocin production in *S. pneumoniae* has proven to involve complex and tightly regulated pathways, so too does this relationship appear within *S. mutans*. The discovery of the ComRS pathway not only revealed the proximal regulator of SigX in *S. mutans*, but also emphasized the critical importance of growth medium on the phenomenon of competence induction [31]. Recent studies have described two distinct patterns in SigX activation in response to the two known competence-inducing pheromones, XIP and MIP. Spontaneous activation of SigX during the onset of stationary phase occurs as a result of endogenous production of XIP when cells are grown in a chemically defined medium (CDM) that is rich in amino acids but devoid of nutrient peptides [62]. Likewise, SigX in *S. mutans* growing in exponential phase can be stimulated with low amounts (0.01 μM) of exogenously provided XIP. However, an XIP response does not occur in peptide-rich media (where peptides are added as nutrient supplements from sources like peptone), unless high concentrations (>1 µM) of XIP are provided exogenously [79]. It is thought that XIP must compete with non-specific peptides for entry to the cell through the Opp/Ami peptide transporter, and therefore peptide-rich media inhibit this process [69,79]. Conversely, mutacin-inducing peptide (MIP, previously referred to as CSP; see Table 1) causes SigX induction when cells are grown in peptide-rich media, but does not result in observable expression of SigX in CDM. The failure of MIP to induce SigX in CDM cannot be attributed to inactive pheromone, as MIP treatment does activate expression of the bacteriocin CipB (Mutacin V/NlmC), via the activity of BlpR (the *S. pneumoniae* ComE homolog, Table 1) [25]. Notably, while *cipB* transcription was observed, Reck et al. did not detect bacteriocin activity by *S. mutans* cells when grown in CDM and stimulated with exogenous MIP. The reason for this apparent lack of activity is not known, but was proposed that media-dependent post-transcriptional and/or post-translational mechanisms might account for this phenomenon [25].

Most perplexing to studies that have strived to describe MIP and XIP stimulatory responses is that the MIP-induced SigX response requires an intact *comS*, even though exogenous addition of XIP appears ineffective in peptide-rich conditions [79]. Numerous past studies are also consistent with the perceived linkage between competence and bacteriocin pathways [22,24,64,68,71,80,81]. Among these was a confounding observation, where in using a P*_sigX_*::*gfp* (green fluorescent protein) reporter, Aspiras et al. [82] described a bimodal pattern of *sigX* induction in cells growing in a biofilm, suggesting an apparent bimodal regulation of SigX by MIP. With these obvious overlaps between MIP- and XIP-mediated effects, there has been much interest in dissecting the relationship between the bacteriocin and competence quorum sensing pathways in *S. mutans*. 

One of the first studies aimed at untangling these pathways combined GFP reporter activity with fluorescence-activated cell sorting (FACS) to allow for analyzing the response of a population to MIP at the individual cell level [59]. Briefly, an *S. mutans* culture expressing a P*_sigX_*::*gfp* reporter was grown in the presence of 0.2 µM MIP in peptide-rich medium for ~2–2.5 h before the population was observed to display two modes of GFP expression and were accordingly sorted into low and high *sigX*-expressing populations. The GFP transcriptional reporter was fused directly downstream of the promoter of interest, in this case P*_sigX_*, to monitor *sigX* induction. As predicted by previous results indicating bimodal expression patterns, this method yielded two distinct populations of SigX-expressing cell where 30%–50% of cells expressed P*_sigX_::gfp* when stimulated with MIP when compared to undetectable expression in unstimulated cells [22,59,82]. Strikingly, microarray analysis of each subpopulation revealed that genes required for competence (i.e., *comR*, *comS*, *sigX* and the SigX-regulated transformasome genes) were enriched only within the SigX-expressing population. Moreover, bacteriocin-related genes, including *cipB*, were expressed at similar levels across the two populations [59]. By using FACS, Lemme et al. [59] demonstrated population heterogeneity within MIP-stimulated cells for the first time. However, the source(s) of the apparent bimodal expression patterns had yet to be resolved [12].

In another study, Son et al. investigated the effect of MIP and XIP treatment on *S. mutans* cells grown in peptide-rich medium, chemically defined medium, or varying ratios in combination under flow conditions and added new insight into the intricacy of SigX regulation while also confirming key findings reported by Lemme et al. [59,79]. Notably, *S. mutans* grown in FMC (a variant of CDM) and exposed to MIP did not exhibit detectable levels of SigX expression. However, this effect could be reversed upon addition of non-specific peptides (as little as 0.8% *v/v* peptide-rich media into FMC), resulting in bimodal expression of SigX [79]. Bimodality required an intact *comS* locus but did not require *opp*, which is essential for SigX expression in CDM. This surprising result led the authors to propose that in peptide-rich media, ComS can bypass the need to exist extracellularly, and instead can activate SigX without a need to act as an intercellular signal, supporting a model in which bimodality is established through positive feedback of internal ComS on ComR signaling [79]. This proposition questions whether ComRS actually serves as a quorum-sensing system in peptide-rich medium. 

## 9. Steps towards Resolving the Link between BlpRH and ComRS in *S. mutans*

Single cell analyses have thus provided data that question the interconnections between bacteriocin and competence pathways in *S. mutans*. Though MIP-induced SigX expression is clearly occurring in peptide-rich conditions, the genetic link connecting the two pathways at this level remains elusive. Although the mechanism employed by the BlpHR pathway to influence ComRS, and thereby SigX, activity is undefined, it is now understood that SigX does have a direct impact on BlpHR signaling. It was demonstrated both by Reck et al. and Son et al. [25,83] that a newly identified cin-box located immediately upstream of BlpR was required for SigX-mediated expression of the BlpRH locus and this effect was dependent upon XIP-signaling (Figure 2). This regulation of BlpR by SigX clarifies the observation that BlpR expression is up-regulated in the competent subpopulation analyzed by Lemme et al. [59].

However, the BlpHR pathway also affects ComRS signaling. Additional experiments from the Reck et al. [25] study demonstrated that CipB (SMU.1914c/NlmC/Mutacin V) activity is essential for a MIP-induced SigX activation in rich medium conditions, replicating previous findings by Hale et al., Perry et al., and Dufour et al. [22,23,25,71]. This requirement for CipB appears to provide a critical piece of the puzzle as to why *comS* is required for MIP-induced bimodal expression of SigX in peptide-rich conditions and several theories have been proposed to explain this requirement.

As *cipB* encodes a putative bacteriocin, Reck et al. [25] have predicted that CipB exerts its effects via pore formation, and that CipB pores might facilitate entry of XIP independently of Opp, thus overcoming competition for entry generated by non-specific peptides in peptide-rich media. Another mechanism was suggested by Dufour et al. [71] that CipB functions intracellularly to promote the induction of competence at the transcriptional level. In their study, a *cipB*-deficient strain, grown in co-culture with the wild type strain under MIP-inducing conditions (and presumably capable of producing extracellular CipB and an ability to generate pores), was unable to exhibit an increase in transformation efficiency. Further supporting this model, microarray data showed significant decreases in expression of competence-related genes (i.e., *sigX*, *comR* and late-competence genes) in the *cipB*-deficient strain compared to wild type when stimulated by MIP [71].

Alternatively, secreted CipB may elicit *comS*-dependent SigX induction by mechanisms alternative to pore formation. For instance, the mechanism of action for the type AII lantibiotic salivaricin B, a Class I bacteriocin, was recently proposed to exert its effect by binding to the bacterial plasma membrane and inducing the intracellular accumulation of a bacterial cell wall precursor, resulting in cell wall thinning and atypical septal formation without penetration of the membrane [84]. This novel mechanism of action is divergent from other type AII lantibiotics, which are commonly thought to elicit bactericidal effects via pore formation [47]. By employing more direct approaches, such as those used by Barbour et al. to uncover the mechanism of action of salivaricin B, researchers may be able to determine the underlying mechanism for CipB’s requirement for *S. mutans* to become competent in peptide-rich media conditions. 

As mentioned previously, Reck et al. [25] also observed that CipB expression was up regulated in response to MIP, even in CDM. However, no bacteriocin activity was detected from MIP-induced *S. mutans* cells in a bacteriocin overlay assay using *Streptococcus sanguinis* and *Lactococcus lactis* as indicator strains on CDM agar plates. Additionally, Reck et al. demonstrated *cipB* transcriptional expression does in fact occur upon MIP treatment of *S. mutans* growing in CDM liquid culture. These results suggest that CipB, if secreted, along with other known bacteriocins, appear to lack bactericidal activity in CDM conditions, yet these proteins are still transcribed (at least in the case of CipB) in response to MIP-mediated BlpRH activity [25]. It remains to be established whether the apparent lack of bacteriocin activity by *S. mutans* grown in CDM is physiologically relevant or if this phenotype is an artifact of growth in these conditions. Finally, in CDM growth conditions, Reck et al. observed BlpR-dependent activation of CipB in a *blpH*-deficient strain, suggesting that BlpR might receive input from an unknown non-cognate histidine kinase [25]. Alternatively, this observation could be interpreted as non-specific phosphorylation of BlpR, in the absence of the de-phosphorylation activity provided by BlpH, resulting in hyperactive BlpR~P (reviewed by Siryaporn and Goulian [85]).

## 10. Additional Inputs Regulating Competence in *S. mutans*

Even as the intricacies of competence signaling by the known direct regulators (i.e., ComR and SigX) are still under investigation, multiple additional QS pathways have been shown to modulate natural transformation in *S. mutans*. Examples of these QS systems include the CiaRH TCST, important for Mutacin I activity, tolerance to oxidative stress, acid tolerance, and biofilm formation [72,73,74]; the HdrRM locus, involved in Mutacin IV expression [75]; and the RcrRPQ operon, implicated in acid tolerance and regulation of intracellular (p)ppGpp pools [76,77,86] (Figure 2). Cumulatively, the effect of these pathways on competence strongly suggests the importance of *S. mutans*’ ability to integrate environmental cues to effectively undergo natural transformation and bacteriocin expression. Expression of the entire set of transformasome-related genes is an energetically costly endeavor. Similarly, in *S. pneumoniae*, additional input pathways contribute to competence regulation, including the pneumococcal ortholog of the CiaRH TCST [87] and the luxS QS pathway important for iron-dependent biofilm formation [88]. Therefore, *S. mutans* and *S. pneumoniae* appear to have evolved complex sensory mechanisms to maximize the benefit of acquiring eDNA. In the context of these organisms’ lifestyle within the oral and nasopharyngeal cavities, these mechanisms might provide competitive advantages against other commensals and/or pathogens by allowing the coordinated expression of bacteriocins to lyse competitors, yielding a valuable source of eDNA.

## 11. Uncovering Conservation Patterns of Competence and Bacteriocin QS Pathways in *Streptococcus*: Further Indications of Regulatory Interconnectedness?

As additional Streptococcal genomes are sequenced, unveiling putative competence pathways (i.e., ComCDE, ComRS Type I, II, and III), more opportunities have arisen to investigate the potential of natural transformation in these species. For example, the identification of the ComRS pathway in *S. mutans* and *Streptococcus thermophilus* facilitated the discovery of orthologs in *S. pyogenes*, *Streptococcus infantarius*, *Streptococcus macedonius*, and *Streptococcus suis* [89,90,91]. While each of these species expresses SigX in response to treatment with the respective XIP, species within the Pyogenic group, exemplified by *S. pyogenes*, have failed to transform under these conditions [89]. However, when *S. pyogenes* was grown as a biofilm on human keratinocytes, in the absence of exogenous XIP, natural transformation was observed, albeit at low efficiency [92]. These results suggest that unknown environmental cues or signals may provide a yet-to-be identified regulatory input into the pyogenic SigX regulon, possibly exerting a suppressive effect on the development of competence [89].

As an alternative explanation, the ComRS pathway may have evolved a separate function in the Pyogenics. This scenario is unlikely, as ComRS activity was demonstrated to directly activate SigX transcription and this conserved alternative sigma factor is the main effector of competence in all known transformable *Streptococcus* species [15,31,60,89,90,93]. However, it is prudent to consider the possibility that homologous pathways operating upstream of SigX in one species may have diverged in function in another species, best represented by the ComC-regulated QS pathway of *S. pneumoniae* and the homologous pathway in *S. mutans* regulated by MIP. Recognition of such potential caveats, discussed in detail in the review by Johnston et al. would help to discourage the inclination to name a gene based on homology and preliminary phenotypes alone [4]. The resulting surplus of names for an individual gene in *S. mutans* has created confusion, unnecessarily biasing how researchers new to the field approach their QS pathway of interest. For example, the gene product encoded in locus SMU.1914c, predicted to belong to the type II family of bacteriocins, has been referred to in the literature as competence-induced peptide B (CipB) [22], Mutacin V [23], bacteriocin *Streptococcus mutans* A (BsmA) [24], and non-lantibiotic mutacin C (NlmC) [23]. Maintaining a consistent nomenclature, as well as including specific loci identifiers (i.e., strain specific loci IDs) when reporting gene names, might alleviate some of this confusion (see Table 1).

Understanding the regulation of competence pathways has empowered researchers to genetically manipulate, under laboratory conditions, various species of *Streptococcus* including *S. mutans*, *S. thermophilus*, and *S. pneumoniae* [7,31,60]. This advance has greatly benefited the study of these organisms in contexts other than competence, by facilitating relatively rapid gene deletions and/or modifications. As many important pathogenic *Streptococcus* species encode putative competence pathways, yet are currently non-transformable under laboratory conditions, elucidating the conditions to promote natural transformation in these organisms could greatly enhance their rate of genetic investigation.

Recently, *comR* genes of the Type-II ComRS QS pathways (conserved amongst the mutans, bovis, and pyogenic streptococcal groups) were expressed in an *S. mutans* background to demonstrate that these ComR variants elicited SigX activity upon stimulation with XIP. The synthetic peptides added corresponded to a predicted mature XIP encoded near the C-terminus of each genome’s putative *comS.* In this way, productive ComR/XIP activity was confirmed in *S. pyogenes*, and *S. suis* [66,89,91]. Interestingly, previously unstudied ComRs encoded in *Streptococcus agalactiae*, *Streptococcus dysgalactiae*, and two Bovis group isolates exhibited SigX reporter activity in a dose-responsive manner when stimulated with the predicted cognate XIP. These results suggest that ComRS may function to regulate SigX within these organisms’ respective genome [66]. While these preliminary results may seem promising, it is possible that unknown regulatory mechanisms are in place, similar to what is predicted in *S. pyogenes*, impeding the demonstration of natural transformation in these species [89].

To further the understanding of what is required for the development of competence in *Streptococcus*, an extensive report by Khan et al. [94] proposed a defined core gene set required for natural transformation in *S. mutans*. In this study, six new tiling microarray data sets were analyzed, compared, and reconciled with five previously published transcriptome profiles. The authors improved upon the past microarray experiments by using a high density tiling array, by controlling for potential antisense artifacts, and by preparing samples from early log-phase cultures to minimize potential metabolic effects on signaling [94]. As a result of their meticulous methodology, Khan et al. were able to define the BlpH, ComR, and SigX regulons as discrete sets of transcripts [94]. Additionally, this study confirmed previous findings that ComE is directly regulated by SigX [25,83]. Finally, and most importantly, the authors aligned the predicted SigX regulon of five additional *Streptococcus* species (*S. pneumoniae*, *Streptococcus gallolyticus*, *Streptococcus sanguinus*, *S. pyogenes*, and *S. thermophilus*) to that of *S. mutans* to clearly compare gene expression levels between species. This broad comparison allowed for the identification of a pan-streptococcal core gene set regulated by SigX, potentially facilitating the identification of species-specific regulatory pathways governing the transition to the competent state [94].

## 12. Conclusions

The capacity to induce competence under laboratory conditions has proven to be a powerful tool to genetically modify selected species within the genus *Streptococcus.* However, the complexities of this QS pathway differ, even between closely related species (i.e., *S. mutans* and *S. pneumoniae*), underscoring the importance of considering the environmental inputs affecting competence development. As suggested by extensive conservation of these pathways, this ability may apply to an extensive number of species implicated in human, zoonotic, or environmental colonization and/or infection [94]. Furthermore, the emerging interconnectedness between bacteriocin production and competence formation emphasizes the role of natural transformation in a polymicrobial context. Caution should be taken when investigating homologous pathways in related Gram-positive organisms to minimize the effect of bias in the interpretation of gene function (as discussed in the review by Johnston et al. [4]). The prospect of artificially modifying the behavior of bacteria by manipulating QS pathways (i.e., competitive pheromone antagonists) is an exciting endeavor. However, this possibility depends upon a comprehensive understanding of the genes involved.

## Figures and Tables

**Table 1:** Nomenclature of selected genes from the *Streptococcus mutans* UA159 bacteriocin and competence quorum sensing pathways.

*S. mutans* Locus ID	Gene Product Name(s)	Function	References
SMU.1914c	CipB, Mutacin V, NlmC, BsmA	Type II bacteriocin	[22,23,24]
SMU.1915	MIP, ComC/CSP	Type II bacteriocin/pheromone	[25,26]
SMU.286	NlmT, ComA	Bacteriocin transporter	[11,27,28]
SMU.287	NlmE, ComB	NlmT accessory factor	[11,27,28]
SMU.1916	BlpH, ComD	Histidine kinase	[26,29]
SMU.1917	BlpR, ComE	Response regulator	[26,29]
SMU.1997	SigX, ComX	Alternative sigma factor	[30,31]
SMU.61	ComR	Type-II ComR; Transcriptional regulator of competence	[31]
N/A	ComS	Pre-peptide of XIP pheromone	[31]
SMU.1897-.1898	CslA, ComA	ABC transporter ATP-binding protein/permease	[30,32]
SMU.1900	CslB, ComB	ABC transporter accessory protein	[30,32]
SMU.381c	Smu.381c	Type-I ComR; Putative transcriptional regulator	[31]

Gene names in bold are the designations used in this review and are based on the current understanding of the gene product’s function in *S. mutans*. The species-specific locus IDs are included for further clarification. Alternative names proposed for each gene are listed following the designated names. References cited correspond to the original report(s) referencing each locus with either the designated or alternative name(s).

## References

[1] Griffith F. (1928). The Significance of Pneumococcal Types. J. Hyg. (Lond).

[2] Papenfort K., Bassler B.L. (2016). Quorum sensing signal-response systems in Gram-negative bacteria. Nat. Rev. Microbiol..

[3] Monnet V., Juillard V., Gardan R. (2014). Peptide conversations in Gram-positive bacteria. Crit. Rev. Microbiol..

[4] Johnston C., Martin B., Fichant G., Polard P., Claverys J.P. (2014). Bacterial transformation: distribution, shared mechanisms and divergent control. Nat. Rev. Microbiol..

[5] Perez-Pascual D., Monnet V., Gardan R. (2016). Bacterial Cell-Cell Communication in the Host via RRNPP Peptide-Binding Regulators. Front. Microbiol..

[6] Mell J.C., Redfield R.J. (2014). Natural competence and the evolution of DNA uptake specificity. J. Bacteriol..

[7] Havarstein L.S., Coomaraswamy G., Morrison D.A. (1995). An unmodified heptadecapeptide pheromone induces competence for genetic transformation in *Streptococcus pneumoniae*. Proc. Natl. Acad. Sci. USA.

[8] Eijsink V.G., Axelsson L., Diep D.B., Havarstein L.S., Holo H., Nes I.F. (2002). Production of class II bacteriocins by lactic acid bacteria; an example of biological warfare and communication. Antonie Leeuwenhoek.

[9] Håvarstein L.S., Holo H., Nes I.F. (1994). The leader peptide of colicin V shares consensus sequences with leader peptides that are common among peptide bacteriocins produced by Gram-positive bacteria. Microbiology.

[10] Havarstein L.S., Diep D.B., Nes I.F. (1995). A family of bacteriocin ABC transporters carry out proteolytic processing of their substrates concomitant with export. Mol. Microbiol..

[11] Hossain M.S., Biswas I. (2012). An extracelluar protease, SepM, generates functional competence-stimulating peptide in *Streptococcus mutans* UA159. J. Bacteriol..

[12] Havarstein L.S., Gaustad P., Nes I.F., Morrison D.A. (1996). Identification of the streptococcal competence-pheromone receptor. Mol. Microbiol..

[13] Pestova E.V., Havarstein L.S., Morrison D.A. (1996). Regulation of competence for genetic transformation in *Streptococcus pneumoniae* by an auto-induced peptide pheromone and a two-component regulatory system. Mol. Microbiol..

[14] Martin B., Soulet A.L., Mirouze N., Prudhomme M., Mortier-Barriere I., Granadel C., Noirot-Gros M.F., Noirot P., Polard P., Claverys J.P. (2013). ComE/ComE~P interplay dictates activation or extinction status of pneumococcal X-state (competence). Mol. Microbiol..

[15] Lee M.S., Morrison D.A. (1999). Identification of a new regulator in *Streptococcus pneumoniae* linking quorum sensing to competence for genetic transformation. J. Bacteriol..

[16] Martin B., Granadel C., Campo N., Henard V., Prudhomme M., Claverys J.P. (2010). Expression and maintenance of ComD-ComE, the two-component signal-transduction system that controls competence of Streptococcus pneumoniae. Mol. Microbiol..

[17] Lin J., Zhu L., Lau G.W. (2016). Disentangling competence for genetic transformation and virulence in *Streptococcus pneumoniae*. Curr. Genet..

[18] Fontaine L., Wahl A., Flechard M., Mignolet J., Hols P. (2015). Regulation of competence for natural transformation in streptococci. Infect. Genet. Evol..

[19] Peterson S., Cline R.T., Tettelin H., Sharov V., Morrison D.A. (2000). Gene expression analysis of the *Streptococcus pneumoniae* competence regulons by use of DNA microarrays. J. Bacteriol..

[20] Peterson S.N., Sung C.K., Cline R., Desai B.V., Snesrud E.C., Luo P., Walling J., Li H.Y., Mintz M., Tsegaye G. (2004). Identification of competence pheromone responsive genes in *Streptococcus pneumoniae* by use of DNA microarrays. Mol. Microbiol..

[21] Ween O., Gaustad P., Havarstein L.S. (1999). Identification of DNA binding sites for ComE, a key regulator of natural competence in *Streptococcus pneumoniae*. Mol. Microbiol..

[22] Perry J.A., Jones M.B., Peterson S.N., Cvitkovitch D.G., Levesque C.M. (2009). Peptide alarmone signalling triggers an auto-active bacteriocin necessary for genetic competence. Mol. Microbiol..

[23] Hale J.D., Ting Y.T., Jack R.W., Tagg J.R., Heng N.C. (2005). Bacteriocin (mutacin) production by *Streptococcus mutans* genome sequence reference strain UA159: Elucidation of the antimicrobial repertoire by genetic dissection. Appl. Environ. Microbiol..

[24] van der Ploeg J.R. (2005). Regulation of bacteriocin production in *Streptococcus mutans* by the quorum-sensing system required for development of genetic competence. J. Bacteriol..

[25] Reck M., Tomasch J., Wagner-Dobler I. (2015). The Alternative Sigma Factor SigX Controls Bacteriocin Synthesis and Competence, the Two Quorum Sensing Regulated Traits in *Streptococcus mutans*. PLoS Genet..

[26] Li Y.H., Lau P.C., Lee J.H., Ellen R.P., Cvitkovitch D.G. (2001). Natural genetic transformation of *Streptococcus mutans* growing in biofilms. J. Bacteriol..

[27] Hale J.D., Heng N.C., Jack R.W., Tagg J.R. (2005). Identification of *nlmTE*, the locus encoding the ABC transport system required for export of nonlantibiotic mutacins in *Streptococcus mutans*. J. Bacteriol..

[28] Yoshida A., Kuramitsu H.K. (2002). Multiple streptococcus mutans genes are involved in biofilm formation. Appl. Environ. Microbiol..

[29] Martin B., Quentin Y., Fichant G., Claverys J.P. (2006). Independent evolution of competence regulatory cascades in streptococci?. Trends Microbiol..

[30] Li Y.H., Tang N., Aspiras M.B., Lau P.C., Lee J.H., Ellen R.P., Cvitkovitch D.G. (2002). A quorum-sensing signaling system essential for genetic competence in Streptococcus mutans is involved in biofilm formation. J. Bacteriol..

[31] Mashburn-Warren L., Morrison D.A., Federle M.J. (2010). A novel double-tryptophan peptide pheromone controls competence in *Streptococcus spp*. via an Rgg regulator. Mol. Microbiol..

[32] Petersen F.C., Scheie A.A. (2000). Genetic transformation in Streptococcus mutans requires a peptide secretion-like apparatus. Oral Microbiol. Immunol..

[33] Wholey W.Y., Kochan T.J., Storck D.N., Dawid S. (2016). Coordinated Bacteriocin Expression and Competence in *Streptococcus pneumoniae* Contributes to Genetic Adaptation through Neighbor Predation. PLoS Pathog..

[34] Kjos M., Miller E., Slager J., Lake F.B., Gericke O., Roberts I.S., Rozen D.E., Veening J.W. (2016). Expression of *Streptococcus pneumoniae* Bacteriocins Is Induced by Antibiotics via Regulatory Interplay with the Competence System. PLoS Pathog..

[35] Luo P., Li H., Morrison D.A. (2004). Identification of ComW as a new component in the regulation of genetic transformation in *Streptococcus pneumoniae*. Mol. Microbiol..

[36] Sung C.K., Morrison D.A. (2005). Two distinct functions of ComW in stabilization and activation of the alternative sigma factor ComX in *Streptococcus pneumoniae*. J. Bacteriol..

[37] Tovpeko Y., Morrison D.A. (2014). Competence for genetic transformation in *Streptococcus pneumoniae*: mutations in *sigmaA* bypass the *comW* requirement. J. Bacteriol..

[38] de Saizieu A., Gardes C., Flint N., Wagner C., Kamber M., Mitchell T.J., Keck W., Amrein K.E., Lange R. (2000). Microarray-based identification of a novel *Streptococcus pneumoniae* regulon controlled by an autoinduced peptide. J. Bacteriol..

[39] Havarstein L.S., Martin B., Johnsborg O., Granadel C., Claverys J.P. (2006). New insights into the pneumococcal fratricide: relationship to clumping and identification of a novel immunity factor. Mol. Microbiol..

[40] Knutsen E., Ween O., Havarstein L.S. (2004). Two separate quorum-sensing systems upregulate transcription of the same ABC transporter in *Streptococcus pneumoniae*. J. Bacteriol..

[41] Claverys J.P., Martin B., Polard P. (2009). The genetic transformation machinery: composition, localization, and mechanism. FEMS Microbiol. Rev..

[42] Steinmoen H., Knutsen E., Havarstein L.S. (2002). Induction of natural competence in *Streptococcus pneumoniae* triggers lysis and DNA release from a subfraction of the cell population. Proc. Natl. Acad. Sci. USA.

[43] Steinmoen H., Teigen A., Havarstein L.S. (2003). Competence-induced cells of *Streptococcus pneumoniae* lyse competence-deficient cells of the same strain during cocultivation. J. Bacteriol..

[44] Guiral S., Mitchell T.J., Martin B., Claverys J.P. (2005). Competence-programmed predation of noncompetent cells in the human pathogen *Streptococcus pneumoniae*: genetic requirements. Proc. Natl. Acad. Sci. USA.

[45] Cotter P.D., Hill C., Ross R.P. (2005). Bacteriocins: developing innate immunity for food. Nat. Rev. Microbiol..

[46] Diep D.B., Skaugen M., Salehian Z., Holo H., Nes I.F. (2007). Common mechanisms of target cell recognition and immunity for class II bacteriocins. Proc. Natl. Acad. Sci. USA.

[47] Alvarez-Sieiro P., Montalban-Lopez M., Mu D., Kuipers O.P. (2016). Bacteriocins of lactic acid bacteria: extending the family. Appl. Microbiol. Biotechnol..

[48] Sanchez-Puelles J.M., Ronda C., Garcia J.L., Garcia P., Lopez R., Garcia E. (1986). Searching for autolysin functions. Characterization of a pneumococcal mutant deleted in the *lytA* gene. Eur. J. Biochem..

[49] Johnsborg O., Havarstein L.S. (2009). Regulation of natural genetic transformation and acquisition of transforming DNA in *Streptococcus pneumoniae*. FEMS Microbiol. Rev..

[50] Berg K.H., Biornstad T.J., Johnsborg O., Havarstein L.S. (2012). Properties and biological role of streptococcal fratricins. Appl. Environ. Microbiol..

[51] Throup J.P., Koretke K.K., Bryant A.P., Ingraham K.A., Chalker A.F., Ge Y., Marra A., Wallis N.G., Brown J.R., Holmes D.J. (2000). A genomic analysis of two-component signal transduction in *Streptococcus pneumoniae*. Mol. Microbiol..

[52] Son M.R., Shchepetov M., Adrian P.V., Madhi S.A., de Gouveia L., von Gottberg A., Klugman K.P., Weiser J.N., Dawid S. (2011). Conserved mutations in the pneumococcal bacteriocin transporter gene, *blpA*, result in a complex population consisting of producers and cheaters. MBio.

[53] Reichmann P., Hakenbeck R. (2000). Allelic variation in a peptide-inducible two-component system of *Streptococcus pneumoniae*. FEMS Microbiol. Lett..

[54] Dawid S., Roche A.M., Weiser J.N. (2007). The *blp* bacteriocins of *Streptococcus pneumoniae* mediate intraspecies competition both in vitro and in vivo. Infect. Immun..

[55] Prudhomme M., Attaiech L., Sanchez G., Martin B., Claverys J.P. (2006). Antibiotic stress induces genetic transformability in the human pathogen *Streptococcus pneumoniae*. Science.

[56] Slager J., Kjos M., Attaiech L., Veening J.W. (2014). Antibiotic-induced replication stress triggers bacterial competence by increasing gene dosage near the origin. Cell.

[57] Stevens K.E., Chang D., Zwack E.E., Sebert M.E. (2011). Competence in *Streptococcus pneumoniae* is regulated by the rate of ribosomal decoding errors. MBio.

[58] Lange R., Wagner C., de Saizieu A., Flint N., Molnos J., Stieger M., Caspers P., Kamber M., Keck W., Amrein K.E. (1999). Domain organization and molecular characterization of 13 two-component systems identified by genome sequencing of *Streptococcus pneumoniae*. Gene.

[59] Lemme A., Grobe L., Reck M., Tomasch J., Wagner-Dobler I. (2011). Subpopulation-specific transcriptome analysis of competence-stimulating-peptide-induced *Streptococcus mutans*. J. Bacteriol..

[60] Fontaine L., Boutry C., de Frahan M.H., Delplace B., Fremaux C., Horvath P., Boyaval P., Hols P. (2010). A novel pheromone quorum-sensing system controls the development of natural competence in *Streptococcus thermophilus* and *Streptococcus salivarius*. J. Bacteriol..

[61] Khan R., Rukke H.V., Ricomini Filho A.P., Fimland G., Arntzen M.O., Thiede B., Petersen F.C. (2012). Extracellular identification of a processed type II ComR/ComS pheromone of *Streptococcus mutans*. J. Bacteriol..

[62] Desai K., Mashburn-Warren L., Federle M.J., Morrison D.A. (2012). Development of competence for genetic transformation of *Streptococcus mutans* in a chemically defined medium. J. Bacteriol..

[63] Fontaine L., Goffin P., Dubout H., Delplace B., Baulard A., Lecat-Guillet N., Chambellon E., Gardan R., Hols P. (2013). Mechanism of competence activation by the ComRS signalling system in streptococci. Mol. Microbiol..

[64] Okinaga T., Xie Z., Niu G., Qi F., Merritt J. (2010). Examination of the *hdrRM* regulon yields insight into the competence system of *Streptococcus mutans*. Mol. Oral Microbiol..

[65] Gardan R., Besset C., Gitton C., Guillot A., Fontaine L., Hols P., Monnet V. (2013). Extracellular life cycle of ComS, the competence-stimulating peptide of *Streptococcus thermophilus*. J. Bacteriol..

[66] Shanker E., Morrison D.A., Talagas A., Nessler S., Federle M.J., Prehna G. (2016). Pheromone Recognition and Selectivity by ComR Proteins among Streptococcus Species. PLoS Pathog..

[67] Talagas A., Fontaine L., Ledesma-Garca L., Mignolet J., Li de la Sierra-Gallay I., Lazar N., Aumont-Nicaise M., Federle M.J., Prehna G., Hols P. (2016). Structural Insights into Streptococcal Competence Regulation by the Cell-to-Cell Communication System ComRS. PLoS Pathog..

[68] Dufour D., Levesque C.M. (2013). Cell death of *Streptococcus mutans* induced by a quorum-sensing peptide occurs via a conserved streptococcal autolysin. J. Bacteriol..

[69] Gardan R., Besset C., Guillot A., Gitton C., Monnet V. (2009). The oligopeptide transport system is essential for the development of natural competence in Streptococcus thermophilus strain LMD-9. J. Bacteriol..

[70] Kreth J., Hung D.C., Merritt J., Perry J., Zhu L., Goodman S.D., Cvitkovitch D.G., Shi W., Qi F. (2007). The response regulator ComE in Streptococcus mutans functions both as a transcription activator of mutacin production and repressor of CSP biosynthesis. Microbiology.

[71] Dufour D., Cordova M., Cvitkovitch D.G., Levesque C.M. (2011). Regulation of the competence pathway as a novel role associated with a streptococcal bacteriocin. J. Bacteriol..

[72] Ahn S.J., Wen Z.T., Burne R.A. (2006). Multilevel control of competence development and stress tolerance in *Streptococcus mutans* UA159. Infect. Immun..

[73] Qi F., Merritt J., Lux R., Shi W. (2004). Inactivation of the *ciaH* gene in *Streptococcus mutans* diminishes mutacin production and competence development, alters sucrose-dependent biofilm formation, and reduces stress tolerance. Infect. Immun..

[74] Levesque C.M., Mair R.W., Perry J.A., Lau P.C., Li Y.H., Cvitkovitch D.G. (2007). Systemic inactivation and phenotypic characterization of two-component systems in expression of *Streptococcus mutans* virulence properties. Lett. Appl. Microbiol..

[75] Merritt J., Zheng L., Shi W., Qi F. (2007). Genetic characterization of the *hdrRM* operon: a novel high-cell-density-responsive regulator in *Streptococcus mutans*. Microbiology.

[76] Seaton K., Ahn S.J., Burne R.A. (2015). Regulation of competence and gene expression in *Streptococcus mutans* by the RcrR transcriptional regulator. Mol. Oral Microbiol..

[77] Seaton K., Ahn S.J., Sagstetter A.M., Burne R.A. (2011). A transcriptional regulator and ABC transporters link stress tolerance, (p)ppGpp, and genetic competence in *Streptococcus mutans*. J. Bacteriol..

[78] McLuhan M. (1964). Understanding Media; The Extensions of Man.

[79] Son M., Ahn S.J., Guo Q., Burne R.A., Hagen S.J. (2012). Microfluidic study of competence regulation in *Streptococcus mutans*: Environmental inputs modulate bimodal and unimodal expression of *comX*. Mol. Microbiol..

[80] Petersen F.C., Fimland G., Scheie A.A. (2006). Purification and functional studies of a potent modified quorum-sensing peptide and a two-peptide bacteriocin in *Streptococcus mutans*. Mol. Microbiol..

[81] Okinaga T., Niu G., Xie Z., Qi F., Merritt J. (2010). The *hdrRM* operon of *Streptococcus mutans* encodes a novel regulatory system for coordinated competence development and bacteriocin production. J. Bacteriol..

[82] Aspiras M.B., Ellen R.P., Cvitkovitch D.G. (2004). ComX activity of *Streptococcus mutans* growing in biofilms. FEMS Microbiol. Lett..

[83] Son M., Shields R.C., Ahn S.J., Burne R.A., Hagen S.J. (2015). Bidirectional signaling in the competence regulatory pathway of *Streptococcus mutans*. FEMS Microbiol. Lett..

[84] Barbour A., Tagg J., Abou-Zied O.K., Philip K. (2016). New insights into the mode of action of the lantibiotic salivaricin B. Sci. Rep..

[85] Siryaporn A., Goulian M. (2010). Characterizing cross-talk *in vivo* avoiding pitfalls and overinterpretation. Methods Enzymol..

[86] Kaspar J., Kim J.N., Ahn S.J., Burne R.A. (2016). An essential role for (p)ppGpp in the integration of stress tolerance, peptide signaling, and competence development in *Streptococcus mutans*. Front. Microbiol..

[87] Dagkessamanskaia A., Moscoso M., Henard V., Guiral S., Overweg K., Reuter M., Martin B., Wells J., Claverys J.P. (2004). Interconnection of competence, stress and CiaR regulons in Streptococcus pneumoniae: competence triggers stationary phase autolysis of ciaR mutant cells. Mol. Microbiol..

[88] Trappetti C., Potter A.J., Paton A.W., Oggioni M.R., Paton J.C. (2011). LuxS mediates iron-dependent biofilm formation, competence, and fratricide in Streptococcus pneumoniae. Infect. Immun..

[89] Mashburn-Warren L., Morrison D.A., Federle M.J. (2012). The cryptic competence pathway in *Streptococcus pyogenes* is controlled by a peptide pheromone. J. Bacteriol..

[90] Morrison D.A., Guedon E., Renault P. (2013). Competence for natural genetic transformation in the *Streptococcus bovis* group streptococci *S. infantarius* and *S. macedonicus*. J. Bacteriol..

[91] Zaccaria E., van Baarlen P., de Greeff A., Morrison D.A., Smith H., Wells J.M. (2014). Control of competence for DNA transformation in *Streptococcus suis* by genetically transferable pherotypes. PLoS ONE.

[92] Marks L.R., Mashburn-Warren L., Federle M.J., Hakansson A.P. (2014). *Streptococcus pyogenes* biofilm growth in vitro and in vivo and its role in colonization, virulence, and genetic exchange. J. Infect. Dis..

[93] Zaccaria E., Wells J.M., van Baarlen P. (2016). Metabolic Context of the Competence-Induced Checkpoint for Cell Replication in *Streptococcus suis*. PLoS ONE.

[94] Khan R., Rukke H.V., Høvik H., Åmdal H.A., Chen T., Morrison D.A., Petersen F.C., McFall-Ngai M.J. (2016). Comprehensive transcriptome profiles of *Streptococcus mutans* UA159 map core streptococcal competence genes. mSystems.

